# Reduction of In-Stent Restenosis Risk on Nickel-Free Stainless Steel by Regulating Cell Apoptosis and Cell Cycle

**DOI:** 10.1371/journal.pone.0062193

**Published:** 2013-04-26

**Authors:** Liming Li, Shuang Pan, Xiaohang Zhou, Xin Meng, Xiaoxi Han, Yibin Ren, Ke Yang, Yifu Guan

**Affiliations:** 1 Key Laboratory of Medical Cell Biology, Ministry of Education, Department of Biochemistry and Molecular Biology, China Medical University, Shenyang, China; 2 Institute of Biotechnology, College of Sciences, Northeastern University, Shenyang, China; 3 Institute of Metal Research, Chinese Academy of Sciences, Shenyang, China; Villa Torri Hospital, Italy

## Abstract

High nitrogen nickel-free austenitic stainless steel (HNNF SS) is one of the biomaterials developed recently for circumventing the in-stent restenosis (ISR) in coronary stent applications. To understand the ISR-resistance mechanism, we have conducted a comparative study of cellular and molecular responses of human umbilical vein endothelial cells (HUVECs) to HNNF SS and 316L SS (nickel-containing austenitic 316L stainless steel) which is the stent material used currently. CCK-8 analysis and flow cytometric analysis were used to assess the cellular responses (proliferation, apoptosis, and cell cycle), and quantitative real-time PCR (qRT-PCR) was used to analyze the gene expression profile of HUVECs exposed to HNNF SS and 316L SS, respectively. Flow cytometry analysis revealed that 316L SS could activate the cellular apoptosis more efficiently and initiate an earlier entry into the S-phase of cell cycle than HNNF SS. At the molecular level, qRT-PCR results showed that the genes regulating cell apoptosis and autophagy were overexpressed on 316L SS. Further examination indicated that nickel released from 316L SS triggered the cell apoptosis via Fas-Caspase8-Caspase3 exogenous pathway. These molecular mechanisms of HUVECs present a good model for elucidating the observed cellular responses. The findings in this study furnish valuable information for understanding the mechanism of ISR-resistance on the cellular and molecular basis as well as for developing new biomedical materials for stent applications.

## Introduction

In the last two decades, stent implantation has been the first choice in percutaneous coronary interventions (PCI) treatment [1], [2]. The success of this medical innovation has saved a great number of patients. Unfortunately, in-stent restenosis (ISR) has occurred frequently at a ratio as high as 20–30% 6 months after the implantation, which has become a major problem in stent surgical practice [3].

ISR has been characterized by a process called neointimal hyperplasia, a sequential event of inflammation, granulation, extracellular matrix remodeling, and vascular smooth muscle cells (VSMCs) proliferation and migration [4], [5]. Unlike cardiac or skeletal muscle cells, VSMCs are not terminally differentiated rather than being able to continuously modulate their phenotype. In the early stages of tissue fabrication, VSMCs are preferred to be in a synthetic phenotype for accelerating cellular proliferation and matrix secretion needed for tissue generation and maturation. Thereafter, VSMCs must switch to a quiescent and contractile phenotype to mimic the functional properties of the native blood vessel. This latter event is primarily influenced by endothelial cells (ECs). Previous studies on the post-angioplasty follow-ups suggested that, lack of EC layer, VSMCs would acquire a synthetic phenotype, leading to extensive migration, proliferation, and matrix synthesis that contribute to restenosis [5], [6]. In addition, damage to the EC layer during the stent implantation can also lead to neointimal hyperplasia and eventually to ISR [7]. Therefore, to slow down the proliferation of the underlying VSMC while stimulating the proliferation of ECs, the presence of an intact endothelium is a necessary condition for the success of engineered vascular tissues with clinical relevance [8].

The conventional bare-metal stents (BMS) have been modified with a thin layer coating containing particular pharmaceutical agents in the hope of reducing the occurrence of restenosis. The drug-eluting stents (DES) indeed improve the performance of stents to reduce ISR. However, stent thrombosis caused by DES has also been reported, and it has been attributed partially to the impairment of arterial healing process characterized by incomplete re-endothelialization, persistent fibrin deposition and macrophage infiltration in comparison with BMS [9], [10], [11]. To achieve an effective reduction of ISR risk, developing novel metallic material for stent applications has been conducted extensively [2], [12].

Currently, the most commonly used metallic materials for coronary stent implantation is the medical grade 316L stainless steel (316L SS) and cobalt-based alloys such as L605 and MP35N [13]. They did demonstrate many mechanical advantages, but the high nickel content in these metallic materials (usually 10–14%) has been suspected to cause the acute thrombosis and long-term restenosis. This negative outcome has raised concerns from the cardiovascular clinical surgeons as well as stent makers [14], [15], [16], [17] since 316L SS and cobalt-based alloy implants could release metal elements such as iron, cobalt, chromium and nickel due to inevitable corrosions in body environment [18], [19], [20], [21]. Köster et al. suggested that allergic reactions to nickel ions released from stainless steel coronary stents might be one of the triggering mechanisms for the development of ISR [16]. Recently, another study demonstrated that the tissue reaction to the metal components in 316L SS, especially nickel, may play an important role in the CR-ISR (chronic refractory in-stent restenosis) [17].

Many scientists and engineers in the field of material science have devoted a great effort to develop novel types of austenitic stainless steels without nickel element [13], [22], [23], [24]. High nitrogen nickel-free austenitic stainless steel (HNNF SS) has been considered as one of the promising nickel-free stainless steels for medical application, since it possesses attractive mechanical properties, better pitting corrosion resistance and good biocompatibility [12], [22], [23], [24], [25]. Literature search, however, has shown that the effect of the new HNNF SS on the cellular behavior at the molecular level has been much less reported.

In the current study, we have conducted a parallel comparison of the biological effect of the commercial stent material 316L SS and a newly developed HNNF SS. Human umbilical vein endothelial cells (HUVECs) were cultured on 316L SS and HNNF SS, respectively, and the effects of 316L SS and HNNF SS on the cellular responses, including cellular morphology, proliferation, apoptosis and cell cycle, were examined. Furthermore, the expression profiles of several genes which regulate the proliferation and apoptosis of HUVECs were evaluated. The experimental results furnished valuable information to uncover the regulatory mechanisms underlying these cellular responses at the molecular level, and the conclusions obtained in this study will lay a solid foundation for further developing novel biomedical materials for stent applications.

## Materials and Methods

### Materials

The HNNF SS used in this study, 00Cr18Mn15Mo2N (0.5–0.9) developed by Institute of Metal Research, Chinese Academy of Sciences, was melted in a 50 kg pressuring induction melting furnace under the protection of pure nitrogen gas, and its chemical composition was analyzed as C: 0.026; N: 0.62; Cr: 18.62; Mn: 15.8; Mo: 2.78; Si: 0.18; S: 0.004; P: 0.013 wt% and Fe in balance. The 316L SS for comparison, with analyzed composition of C: 0.025; Cr: 17.5; Mn: 1.06; Mo: 2.66; Ni: 13.07; Si: 0.6; S: 0.008; P: 0.02 wt% and Fe in balance, was melted in a 25 kg vacuum induction melting furnace according to ASTM F138-2003. These two steels were forged and rolled to billets for further processing, and a solution treatment was conducted before experiment to obtain a homogeneous austenitic microstructure. All the samples were cut from the rolled materials and were solution treated at 1373 K for 1 h, followed by water quenching, which then were machined into φ32×1 mm or φ12×1 mm slices, respectively, to fit to the 6-well or 24-well culture plates. All the slice samples were grounded with serial SiC papers, electrochemically polished and finally ultrasonically cleaned for 15 min in acetone for three times to remove any impurities on the surface. Prior to experiments, all the samples were ultrasonically cleaned with alcohol, rinsed in distilled water and then sterilized at 121°C for 20 min.

### Cell Culture

HUVECs, purchased from KeyGEN Biotech (Nanjing, China), were maintained in RPMI-1640 medium (Hyclone,Beijing, China) supplemented with 10% fetal bovine serum (FBS) (Invitrogen, Grand Island, USA), 100 IU/ml penicillin and 100 µg/ml streptomycin, in a humidified incubator with 5% CO_2_ at 37°C.

### Cell Adhesion

HUVECs were seeded on surfaces of different steel substrates at a concentration of 1×10^5^ cells/well (24-well plate). After 4-hour incubation, the non-adhered cells were discarded with the medium, and the steel substrates were washed twice with phosphate-buffered saline (PBS) gently. Cells adhered on the substrates were harvested with 0.25% trypsin and the number of cells were counted under an optical microscope with trypan blue. Results were expressed as a percentage of the number with respect to that of the control.

### Observation of Cell Morphology

HUVECs were seeded on the surface of the experimental steel substrates at a density of 1×10^4^ cell/well (24-well plate). Cells were cultured in RPMI-1640 medium containing 10% FBS in a humidified incubator (5% CO_2_ atmosphere at 37°C) for 3-day and 7-day periods without renewing culture medium. After each culture period, the steel substrates were rinsed twice with PBS, and cells on the steel substrates were immediately stained with Giemsa dye (Nanjing Jiancheng Bioengineering Institute, Nanjing, China). The stained cells were examined under an optical microscope (Axiovert 200 MAT, Carl Zeiss, Oberkochen, Germany). Cells cultured in the medium were used as the control and, after the same Giemsa’s staining, were examined under an inverted microscope (Olympus BX-51, Olympus Optical Co., Tokyo, Japan).

For the cell density observation, after cell cultured on steel substrates (24-well plate) for 3 days and 7 days, respectively, 0.5 µM calcein AM (Dojindo Laboratories, Kumamoto, Japan) was added to the medium, and cells were incubated at 37°C for another 30 min. Excess calcein AM on cell surface was removed by several washes with PBS. Images of cells were taken with a fluorescence microscope (Olympus IX-71, Olympus Optical Co., Tokyo, Japan).

### Measurement of Cell Proliferation

To assess the cell viability, HUVECs were seeded on surfaces of different steel substrates at a concentration of 1×10^4^ cells/well (24-well plate), and cultured in an incubator (5% CO_2_ and at 37°C) for 1-, 3-, 5- and 7-day periods. The cell viability was examined using the Cell Counting Kit-8 (CCK-8; Dojindo, Kumamoto, Japan) assay which was based on the principle of CCK-8 (water-soluble tetrazolium salt) cleavage to formazan-class dye by mitochondrial succinate tetrazolium reductase in viable cells. The cell counting solution of 50 µl was added into each well and mixed for 4 h, and then formazan-class dyes were detected by measuring absorbance at 450 nm using a TECAN Infinite M200 microplate reader (Tecan Group Ltd., Maennedorf, Switzerland). Cell viability was expressed as the ratio of the signal obtained from the cells incubated on steel substrates and the cells incubated in culture medium [26].

To assess the cell proliferation directly, the number of cells were counted. HUVECs were seeded on surfaces of different steel substrates at a concentration of 1×10^4^ cells/well (24-well plate). During a 7-day culture incubation, the cells grown on each substrate was harvested at each day and washed with PBS. The number of cells was then counted under an optical microscope with trypan blue.

### Cell Cycle Analysis and Apoptosis Assessment by Flow Cytometry

HUVECs were seeded in a 6-well culture plate at a concentration of 1×10^4^ cells/well and cultured in an incubation with RPMI-1640 medium containing 10% FBS. When cells became confluence, RPMI-1640+10% FBS was replaced with RPMI-1640 medium (without FBS). After serum starvation for 22 h, cells were treated with RPMI-1640+10% FBS for 24 h. At the points of serum starvation for 22 h and serum-retreated for 24 h, cells on the steel substrates were trypsinized, washed with PBS and fixed in ice-cold 70% ethanol overnight. Cells were then treated with RNase at 37°C and stained with propidium iodide (PI) solution (KeyGEN Biotech, Nanjing, China) for 30 min at 4°C. PI-stained nuclei were analyzed with a flow cytometry (FACSCalibur, BD Biosciences, USA). The ratios of the cells in G0/G1, S and G2/M phases were calculated.

For cells apoptotic analysis, HUVECs were seeded in a 6-well culture plate at a concentration of 1×10^4^ cells/well and cultured in an incubator with RPMI-1640 medium +10% FBS for 7 days. Cells were harvested, washed twice with ice-cold PBS and resuspended in the dark with AnnexinV-FITC and PI (KeyGEN Biotech, Nanjing, China) buffer for 15 min at room temperature. Cells were then analyzed with a flow cytometry. Cells were considered to be apoptotic when they were either Annexin V+/PI- (early apoptotic) or Annexin V+/PI+ (late apoptotic). All flow cytometry data were analyzed using the Mod Fit LT software (Verity Software House, Topsham, MN).

### Quantitative Real-time PCR

HUVECs were seeded in a 6-well culture plate at a concentration of 1×10^4^ cells/well and cultured in an incubator with RPMI-1640 medium +10% FBS for 7 days. Total RNA of HUVECs was isolated using TRIzol reagent (Invitrogen, Carlsbad, USA), reverse transcription (RT) was performed with the RT reagent kit (Takara Biotechnology Co., Ltd. Dalian, China), and quantitative real-time PCR (qRT-PCR) was performed using the SYBR Premix Ex Taq II (Takara Biotechnology Co., Ltd. Dalian, China). All these preparations were performed following the manufacturer’s protocols. The qRT-PCR oligonucleotide primers used in this study are shown in Table 1. The amplification and analysis were performed on a Real-Time PCR system (ABI prism 7500, Applied Biosystems, Foster City, USA). The PCR cycle was as follows: 95°C for 30 sec, 40 cycles of 95°C for 5 sec and 60°C for 34 sec and followed by one cycle at 95°C for 15 sec, at 60°C for 1 h and at 95°C for 15 sec. The qRT-PCR data were analyzed with a ΔΔC_t_ method and normalized using NADPH cDNA as an internal control.

**Table 1:** Nucleotide sequences of primers used for qRT-PCR.

Gene	Primer notation	Sequence
GAPDH	Forward	GCACCGTCAAGGCTGAGAAC
	Reverse	TGGTGAAGACGCCAGTGGA
Caspase3	Forward	GACTCTGGAATATCCCTGGACAACA
	Reverse	AGGTTTGCTGCATCGACATCTG
Caspase8	Forward	CATTTGCATATTTAGCCGCCAAG
	Reverse	TTAAGAGTCCCAGGAATTCAGCAAC
Caspase9	Forward	GCCATATCTAGTTTGCCCACACC
	Reverse	CACTGCTCAAAGATGTCGTCCA
Fas	Forward	CAACAACCATGCTGGGCATC
	Reverse	TGATGTCAGTCACTTGGGCATTAAC
Cyclin A1	Forward	GAAATTGTGCCTTGCCTGAGTG
	Reverse	TCTGATATGGAGGTGAAGTTCTGGA
Cyclin D	Forward	ATGTTCGTGGCCTCTAAGATGA
	Reverse	CAGGTTCCACTTGAGCTTGTTC
Cyclin E	Forward	GCCGTTTACAAGCTAAGCAGCAG
	Reverse	CCAGATAATACAGGTGGCCAACAA
Atg5	Forward	TTGAATATGAAGGCACACCACTGAA
	Reverse	GCATCCTTAGATGGACAGTGCAGA
Atg7	Forward	CTGTAACTTAGCCCAGTACCCTGGA
	Reverse	TACGGTCACGGAAGCAAACAAC
Atg12	Forward	AGTAGAGCGAACACGAACCATCC
	Reverse	CCACGCCTGAGACTTGCAGTA

### Statistical Analysis

Data analysis was performed using SPSS software (Chicago, Illinois, USA). All the experimental data were expressed as means±standard deviation (SD), and statistically analyzed. The statistical significance of the results was performed by one-way analysis of variance (ANOVA) followed by Tukey’s post hoc multiple comparison tests. A *p* value less than 0.05 was considered statistically significant. All the experiments were repeated at least for three times.

## Results

### Cell Adhesion

**Figure 1:**
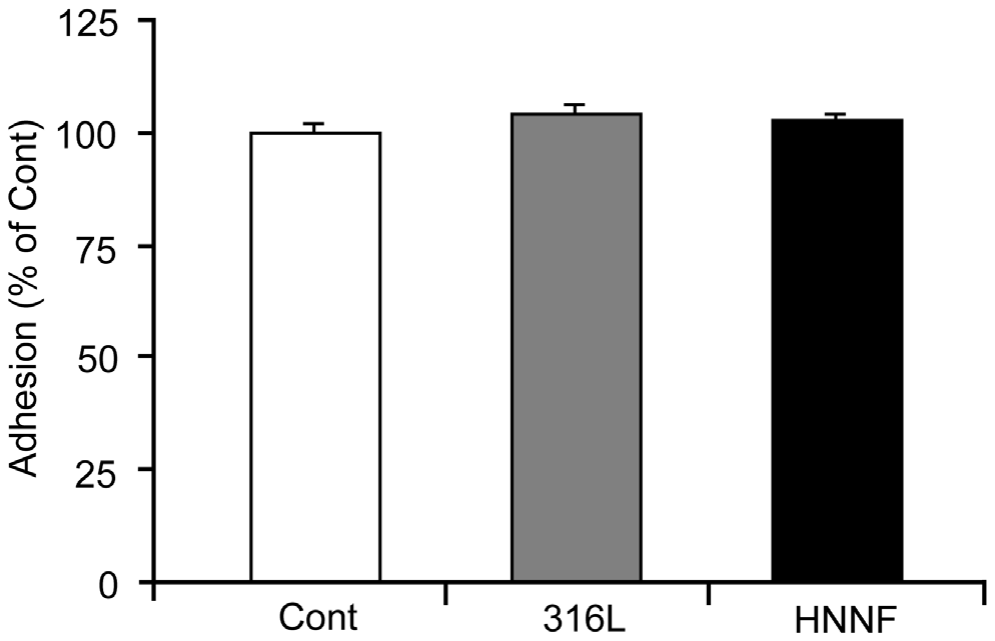
Adhesion of HUVECs to 316L SS substrate, HNNF SS substrate and Type-IV collagen-coated culture dish (referred as control).

To assess the cell adhesion on different surfaces, HUVECs were seeded on different substrates. After cells were cultured for 4 h, cells were harvested and the cell number was counted under microscope by staining with trypan blue. The number of HUVECs grown in Type-IV collagen-coated culture dish with the culture medium was used as control. As shown in Fig. 1, the percentages of cells adhered on surfaces of 316L SS and HNNF SS after 4 h incubation are almost identical to that of the control. No statistical significance (*p*>0.05) was found among different substrates.

### Growth behavior of HUVECs on HNNF SS and 316L SS

**Figure 2:**
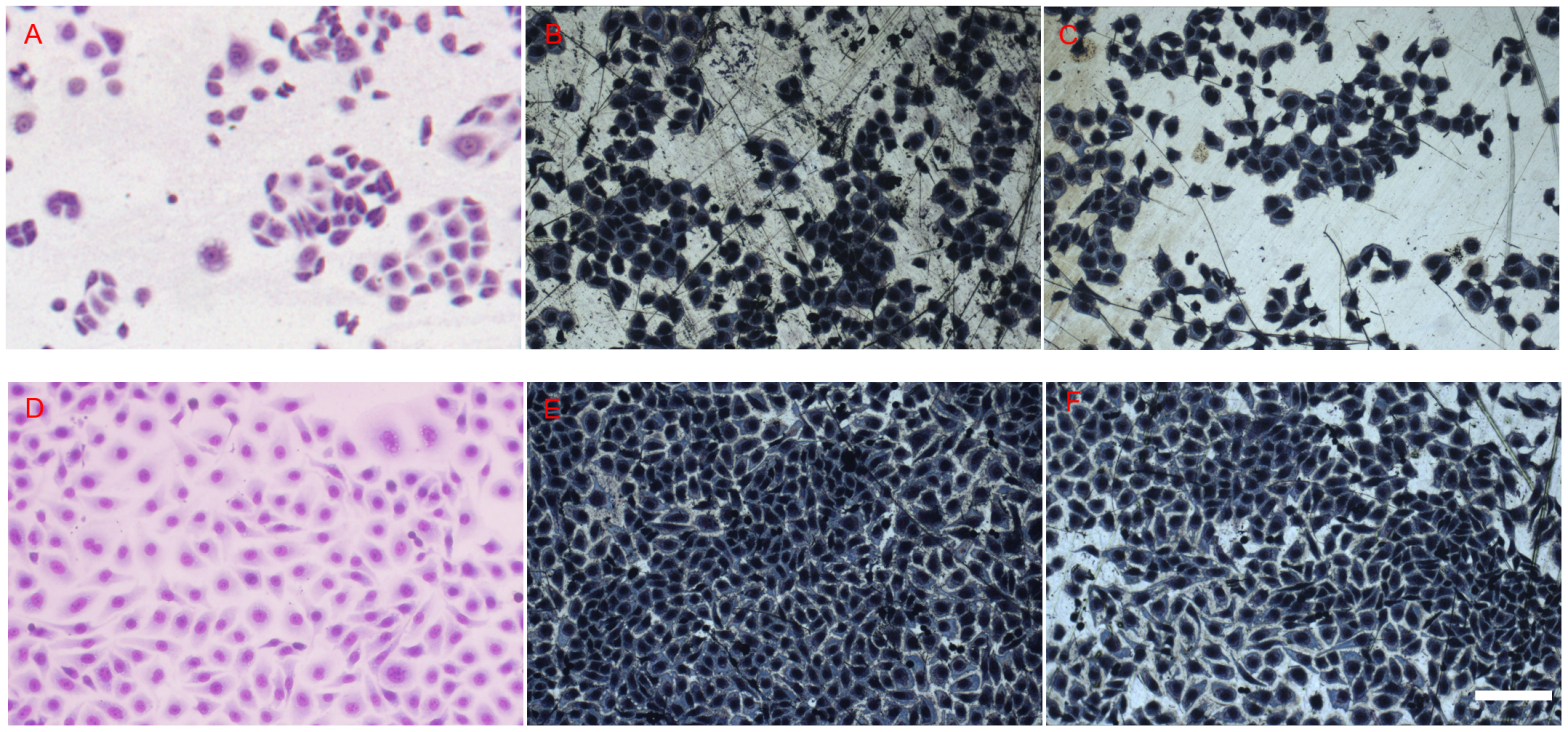
Optical images of HUVECs grown on different surfaces of materials. Culture for 3 days in culture medium (A), 316L SS (B) and HNNF SS (C), and for 7 days in culture medium (D), 316L SS (E) and HNNF SS (F). Scale Bars = 100 µm.

Morphologies of HUVECs cultured on 316L SS and HNNF SS substrates after 3-day and 7-day periods were examined under an optical microscope (Fig. 2). Optical microscopic images show that HUVECs grow uniformly on all the substrate surfaces as a monolayer, no obvious difference in morphology of HUVECs could be identified in comparison with the control. The numbers of cells grown on HNNF SS and 316L SS were increased with the culture time. After 3-day culture, the HUVEC cellular spreading was good on all the evaluated stainless steel substrates, and after 7-day culture, HUVECs covered evenly the entire stainless steel surfaces. The number of HUVECs on 316L SS was higher than that on HNNF SS, and the number of HUVECs on HNNF SS was higher than that of the control.

**Figure 3:**
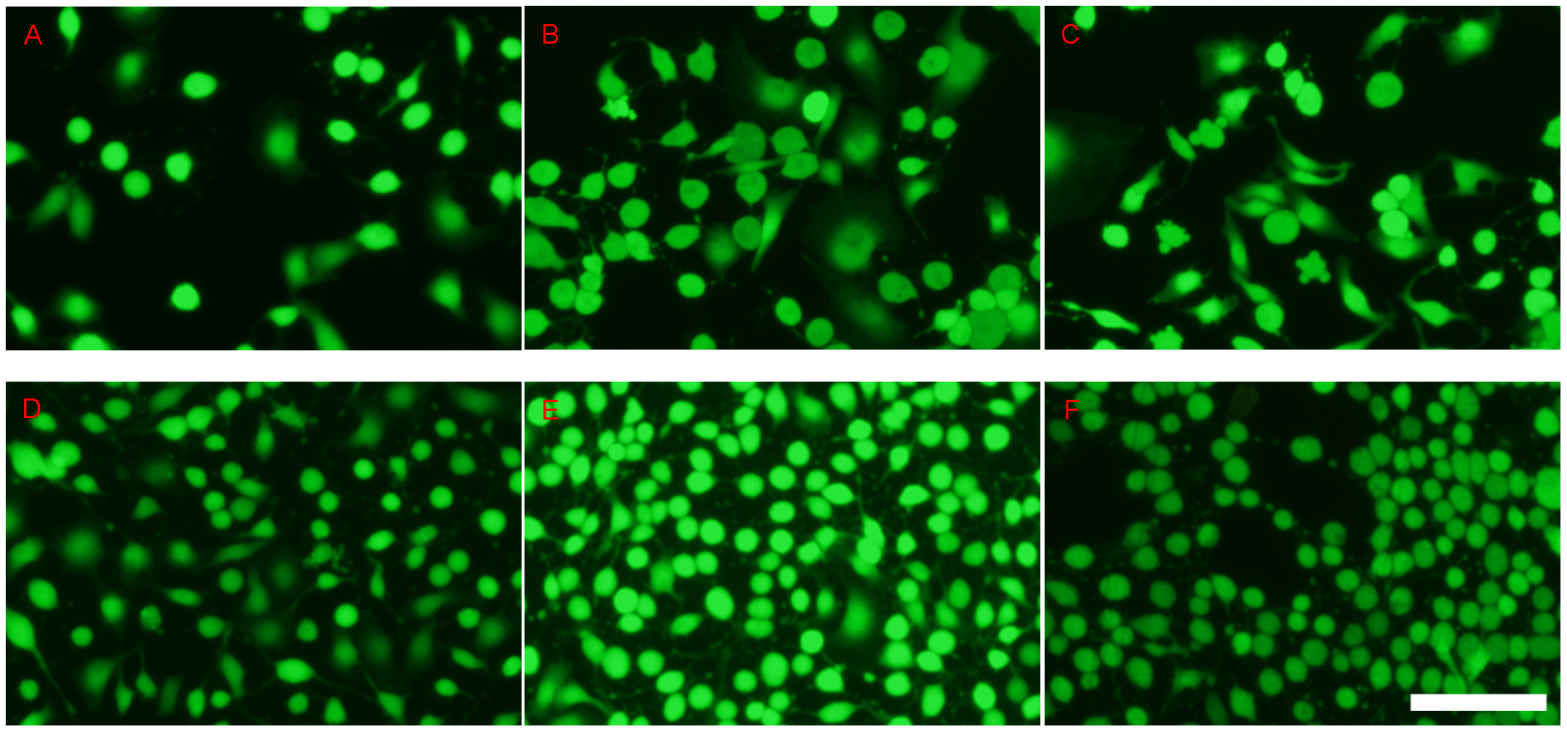
Fluorescent images of HUVECs grown on different surfaces of materials. Culture for 3 days in culture medium (A), 316L SS (B) and HNNF SS (C), and for 7 days in culture medium (D), 316L SS (E) and HNNF SS (F). Scale Bars = 100 µm.

Furthermore, cells on the steel substrates were also stained with calcein AM and their images were recorded under a fluorescence microscope. Calcein-AM is the dye which can stain only the living cells. Fig. 3 shows that the numbers of the living HUVECs on different steel substrates follow the order of 316L SS>HNNF SS>control.

### Cell Proliferation of HUVECs on HNNF SS and 316L SS

**Figure 4:**
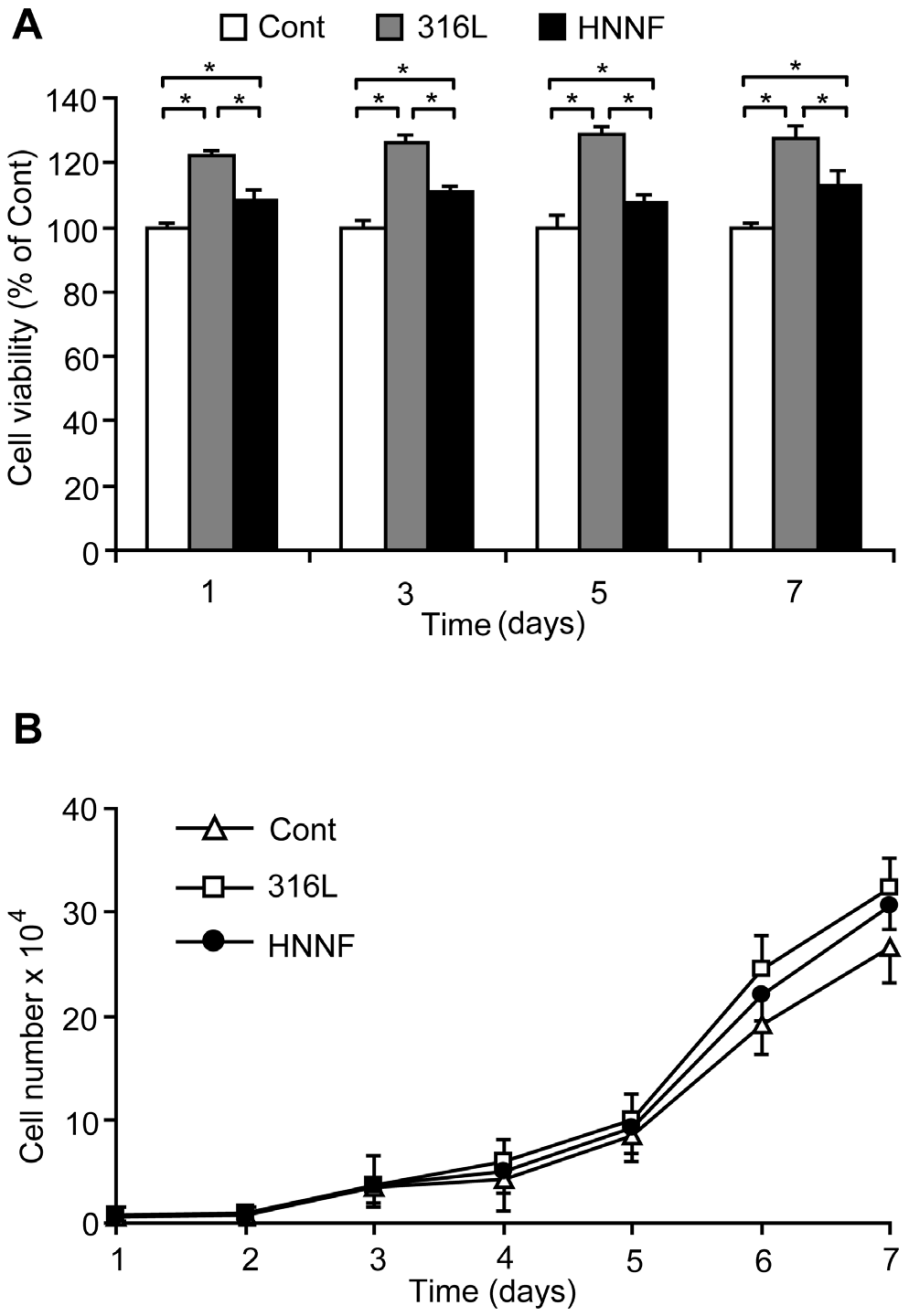
Measurement of HUVEC proliferation. (A) Relative growth rates of HUVECs on 316L SS and HNNF SS after 1-, 3-, 5- and 7-day growth with respect to the control of HUVECs (100%). (B) Growth curve of HUVECs on surfaces of 316L SS and HNNF SS, and in culture medium in a 7-day period.

The proliferation ability of HUVECs on different stainless steel substrates was examined in comparison with the control. HUVECs were seeded on each substrate surface and then incubated in an incubator for 1, 3, 5 and 7 days, and then assayed using CCK-8 method. As shown in Fig. 4a, in comparison with the control, 316L SS substrate shows a higher cell proliferation rate by about 20% in all four incubation periods examined, whereas the cell proliferation rate on HNNF SS is about 10% higher than that of the control.

The cell proliferation behavior was also tested in terms of the absolute cell numbers. During a 7-day period, the absolute numbers of HUVECs grown on different steel substrate were counted and the growth curves were plotted. As illustrated in Fig. 4b, starting from day 3, it became evident that more HUVECs could be observed on 316L SS than on HNNF SS, and the latter was more than the control.

### Effects on Cell Cycle of Synchronized HUVECs

**Figure 5:**
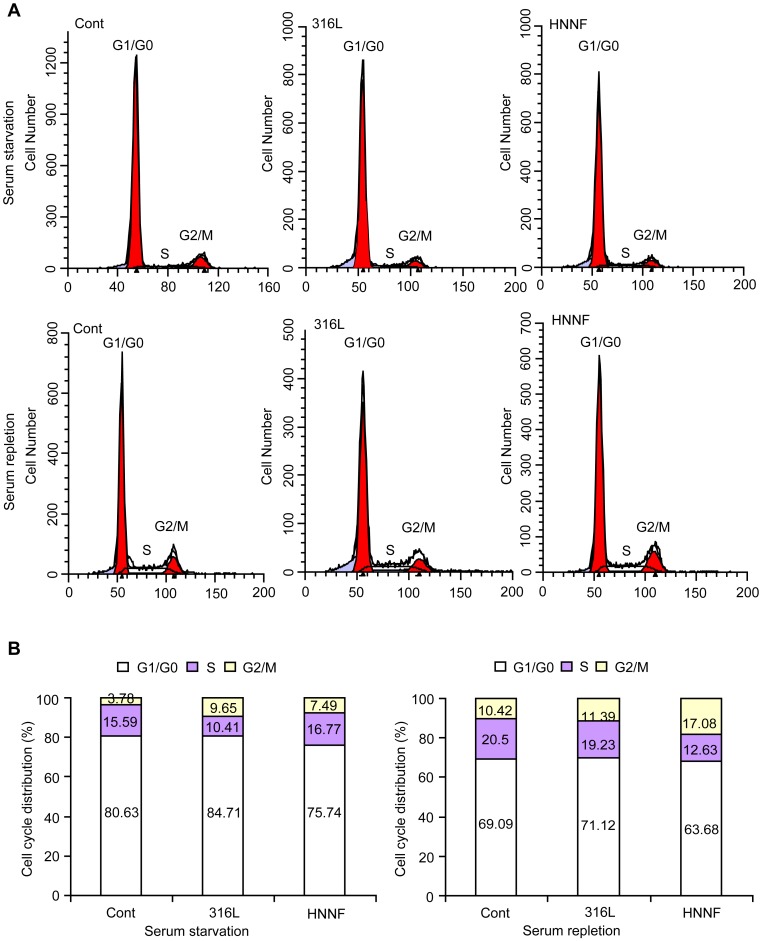
Effect of 316L SS and HNNF SS on cell cycle progression of HUVECs. (A) Flow cytometric data showing the cell cycle distributions of HUVECs cultured on 316L SS and HNNF SS, respectively, without FBS for 22 h (top panel), and cell cycle distributions of HUVECs cultured with the whole culture medium for 24 h after cell synchronization (bottom panel). (B) Histographic representations of the cell cycle distributions of HUVECs.

The influence of 316L SS and HNNF SS on cell cycle progression was analyzed (Fig. 5). The serum-deprivation approach was used to synchronize HUVECs cultured on 316L SS, HNNF SS and in Type-IV collagen-coated culture dish. The serum-deprivation for 22 h has led to 80.63±2.90%, 84.71±2.44% and 75.74±7.99% of HUVECs to be synchronized in the G1/G0 phase for 316L SS, HNNF SS and the control, respectively. Serum-retreatment for 24 h was followed. The cell population in the G1/G0 phase was reduced to 69.09±7.64%, 71.12±10.44% and 63.68±8.33% for 316L SS, HNNF SS and the control, respectively.

### Apoptosis of HUVECs

**Figure 6:**
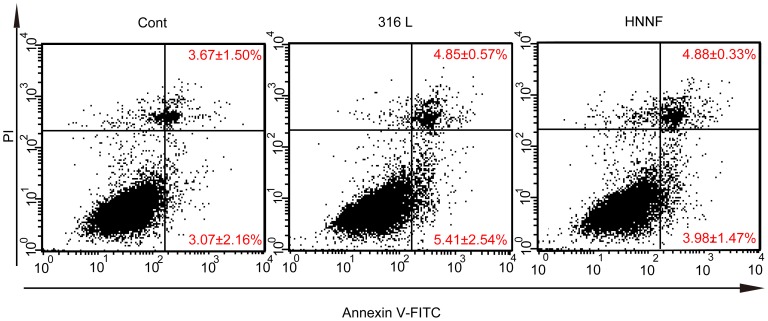
Flow cytometric analysis of apoptosis of HUVECs. Cells were cultured in culture medium, on surfaces of 316L SS and HNNF SS for 7 days.

Flow cytometric analysis was performed to examine the influence of 316L SS and HNNF SS on the apoptotic behavior of HUVECs (Fig. 6). The ratios of the early apoptotic HUVECs were calculated to be 5.41±2.54% and 3.98±1.47% for 316L SS and HNNF SS, respectively, both higher than that of the control (3.07±2.16%). Also, the ratios of the later apoptotic HUVECs were calculated to be 4.85±0.57% and 4.88±0.33% for 316L SS and HNNF SS, respectively, both higher than that of the control (3.67±1.50%).

### Gene Expression Profiles by Quantitative Real-time PCR

**Figure 7:**
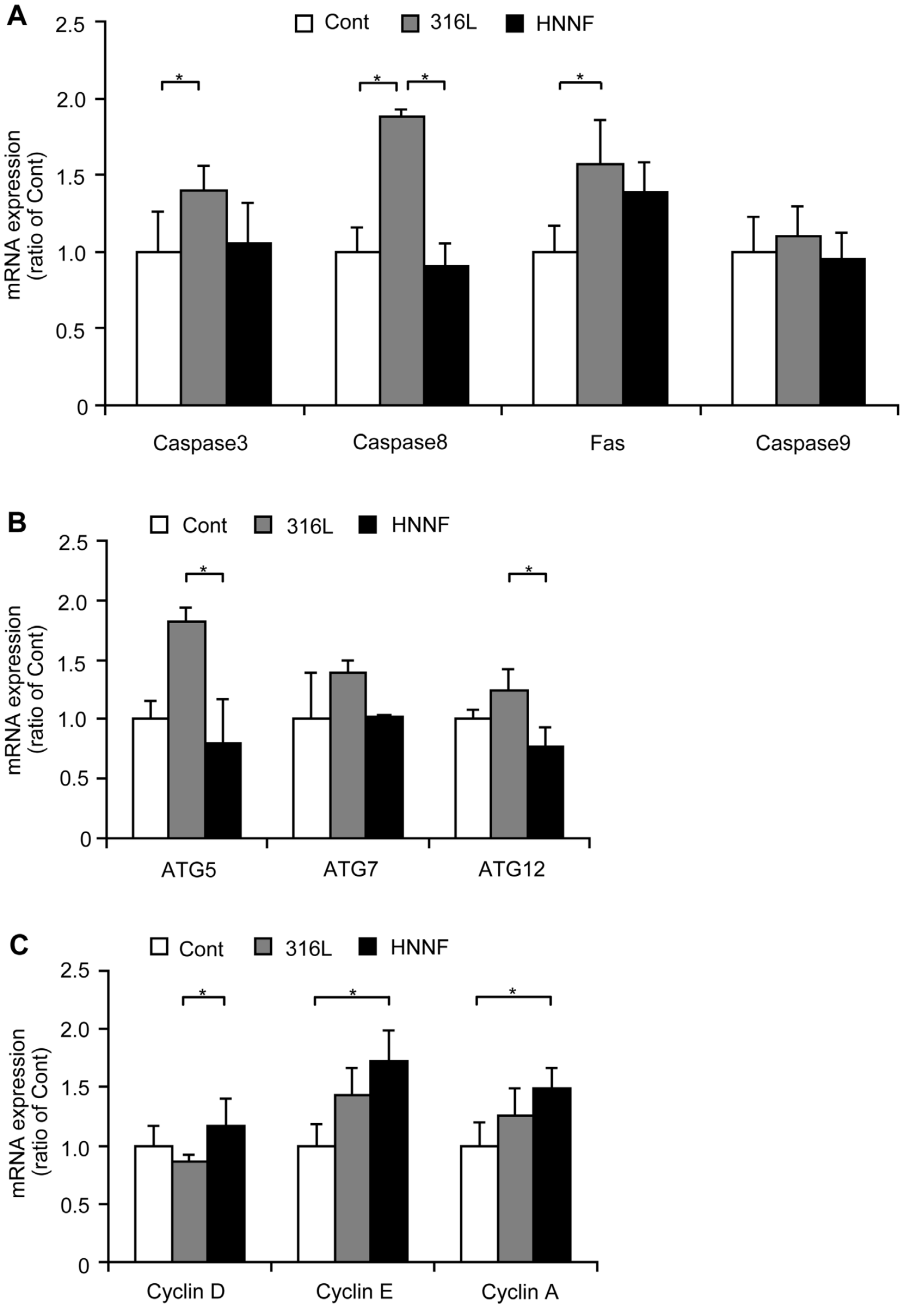
Gene expression profiles of HUVECs. Cells grew in culture medium, on surfaces of 316L SS and HNNF SS for 7 days determined using qRT-PCR method.

As illustrated in Fig. 7, all the genes of caspase-3, 8, 9 and Fas of HUVECs cultured on 316L SS exhibited a notable up-regulated expression, ranging from 10% (caspase-9) to ∼90% (caspase-8). On the other hand, these same genes of HUVECs cultured on HNNF SS were expressed differently: FAS were up-regulated by ∼40%, while others changed slightly within the experimental errors. In all cases, the expressions of these genes examined were always higher on 316L SS than those on HNNF SS.

ATG5, ATG7 and ATG12 of HUVECs on 316L SS substrate also demonstrated an up-regulated expression profile by 80, 40 and 20%, respectively, with respect to the control. However, when cultured on HNNF SS substrate, the expression levels of these genes were just slightly changed within the experimental errors.

Genes participating in the cell cycle also demonstrated different expression profiles. In comparison with the control, Cyclin D, Cyclin E and Cyclin A of HUVECs cultured on HNNF SS substrate were up-regulated by 15, 70 and 45%. In the case of 316L SS, Cyclin E and Cyclin A were up-regulated while Cyclin D was slightly down-regulated.

## Discussion

Many novel biomaterials have been developed in the past two decades in the hope of improving the performance of stent materials. HNNF SS is a newly invented stainless steel and demonstrates a great potential for medical stent-implantation purpose. To evaluate its ISR-resistance capability and feasibility for clinical application, a thorough investigation of the cellular behavior at the molecular level is conducted in comparison with a conventional stent material 316L SS in the present study.

Under a normal physiological condition, a vein wall is composed of two major layers: the inner layer is composed of ECs and the outer layer is composed of smooth muscle cells (SMCs). As an initial step of a systematical study, we focus our attention on HUVECs, because ECs form a flattened monolayer on the elastic basement membrane, and play an important role in maintaining the wall integrity and biological function. These normal EC functions demonstrate an important force in the initial step of vein graft neointimal formation [27].

### Cell Proliferation Properties

We evaluated cell adhesion capability on the different experimental materials by examining cell numbers after 4 h incubation. As illustrated in Fig. 1, there was no statistical significance in the cell adhesion among the different experimental materials.

In order to evaluate the cell response to the different materials more directly, the morphology of HUVECs was examined using optical microscope and fluorescence microscope. As shown in Figs. 2 and 3, the cell number is higher on the 316L SS surface than that on the HNNF SS surface, which, in turn, is higher than that of the control. The images obtained using optical microscope and fluorescence microscope also show a consistence. Meanwhile, the numbers of cells cultured in control medium and on 316L SS and HNNF SS were counted. As the results showed, the number of cells cultured on 316L SS was higher that that on HNNF SS, and both numbers of cells cultured on 316L SS and on HNNF SS were higher than that in control medium (Fig. 4b). The cell viability was also evaluated using CCK-8 assay and results showed a statistical significance between HNNF SS and 316L SS in a 7-day period (Fig. 4a).

The serum deprivation is routinely used for in vitro mitogenicity assays to reduce background proliferation levels and increase the sensitivity [28]. In order to determine the effects of 316L SS and HNNF SS on the progression of the HUVECs through G0/G1 stages of the cell cycle, it is necessary to prepare a cell population in which all the cells should be pre-set at the same phase of the cell cycle. This can be achieved by ‘synchronizing’ HUVEC cell cultures with serum starvation, which arrests cells in the G0/G1 phase [29]. After 22 h serum-deprivation, about 80.6, 84.7 and 75.7% of cell population were arrested in the G0/G1 phase for control, 316L SS and HNNF SS, respectively. Retreated with serum for 24 h, the populations in the G1/G0 phase were reduced to 69.1, 71.1 and 63.7%, equivalent to by 16, 19, and 17%, respectively. These numbers indicate that HUVECs on 316L SS substrate progressed into the S phase more rapidly than that on HNNF SS substrate as well as than the control (Fig. 5). These results are consistent with the result of cell proliferation in this study as well as with the results reported by other studies [30]. Alvarez et al. have conducted a comparative study on the cell growth on 316L SS and nickel-free stainless steels, and found no significant difference in the cell proliferation rate between nickel-free stainless steels and 316L SS. Their results were rather unexpected since the nickel element in 316L SS was considered to possess a significant cytotoxicity potential. In fact, the levels of iron, chromium and nickel ions released into culture medium in a 60-day period were significantly low [30]. Thus, in short periods, these levels on ion release per se might not induce severe cytotoxic effects. As revealed by flow cytometric analysis in Fig. 6, the early apoptotic rate of HUVECs cultured on 316L SS after 7 days became higher than that on HNNF SS, while the late apoptotic rate of cells on 316L SS was no difference in comparison with that on HNNF SS. It could indicate that the cytotoxic effect on HUVECs induced by 316L SS could be observable only after a long period of interaction.

Although from the results of cell morphology, cell viability and cell growth, both 316L SS and HNNF SS demonstrated the ability to stimulate the growth of HUVECs in comparison with the control. However, it seemed that after 7-day incubation, 316L SS might induce more cells apoptosis compared with HNNF SS. Thus, in the extended period of time the released nickel ions (Ni^2+^) might act as cofactors or inhibitors in enzymatic processes involved in protein synthesis and cell proliferation, disrupt intracellular organelles, alter cells morphology, and decrease cells number [30], [31], [32].

For long-term implantation of 316L SS, the negative effect on human body should be considered because of the cytotoxicity and other effects of Ni^2+^ released from the stent material. Previous studies found that even though no cytotoxicity was detected by MTT assay, the expression of massive candidate genes had been dramatically altered [5]. These results suggest that although the cellular response to the nickel toxicity was not obvious in a short interaction period, there has already been a negative effect at the molecular level. It was proposed that the change of gene expression occurred prior to any observable change at the cellular level.

Thus, the gene expression profiles of HUVECs on different substrates were analyzed using qRT-PCR approach. According to the results discussed above, some genes responsible for cell apoptosis and cell cycle have been analyzed, and the potential molecular mechanisms in cells on exposure to 316L SS and nickel-free stainless steels were assessed.

### Apoptotic Properties of HUVECs

**Figure 8:**
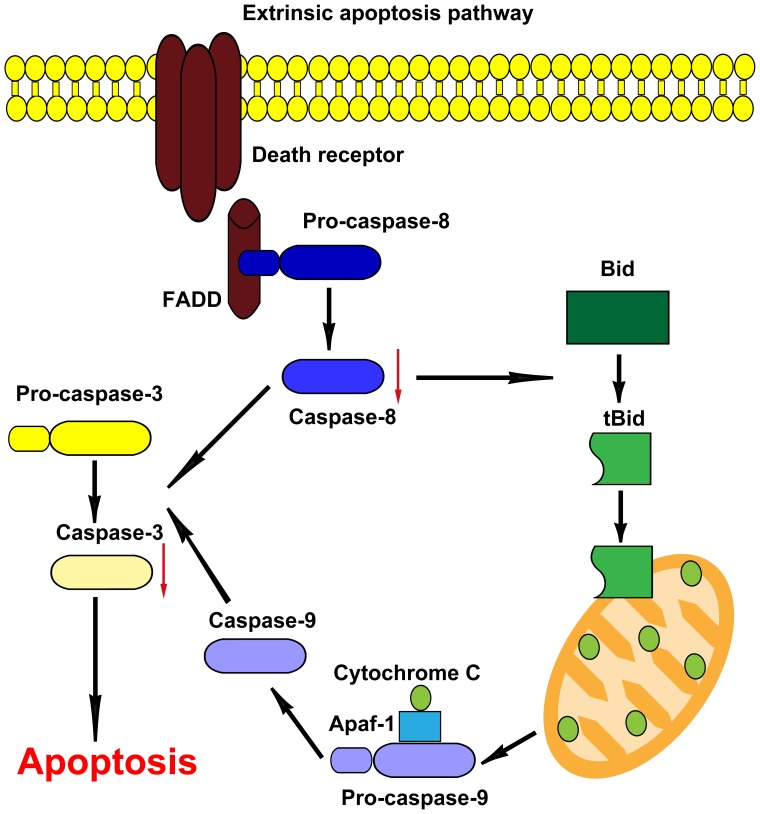
Schematic diagram of pathways proposed in 316L SS and HNNF SS induced cell apoptosis. The proposed model shows that 316L SS and HNNF SS cause cell apoptosis through extrinsic apoptosis pathway by the ligation of Fas ligand to Fas receptor activating caspase-8 and caspase-3, whereas HNNF SS reduces the activation of caspase-8 and caspase-3 compared with 316L SS.

To understand the nature of this apoptotic response to 316L SS, the expression levels of these genes participating in cell apoptosis were further evaluated. Apoptosis has been known to be a natural event occurring in all types of cells, and the apoptotic signaling pathways have been intensively studied during the last decade. As illustrated in Fig. 8, the death-receptor pathway is usually initiated through a binding of a death ligand, such as Fas ligand (FasL), or tumor necrosis factor α (TNF-α) on endogenous death receptors [33]. This binding leads to the recruitment of Fas-associated death domain (FADD), and then activates caspase-8 and subsequent downstream effectors [34]. In the present study, qRT-PCR results showed that gene FAS was highly overexpressed (>50%) on 316L SS and moderately overexpressed (∼40%) on HNNF SS substrates (Fig. 7a). In contrast, the expression of gene caspase-8 was almost doubled on 316L SS whereas it was just at the basal level on HNNF SS. These data indicated that the death receptor pathway has been activated, but it was activated unequally on these two types of metallic materials.

Mitochondrial pathway is another important apoptotic pathway (Fig. 8). This pathway is characterized by the activation of Apaf-1 and caspase-9 [35]. In the current study, the expression level of caspase-9 was almost equal for 316L SS and HNNF SS in comparison with that of the control (Fig. 7a). It might be the sign that the mitochondrial pathway was involved at the basal level in the present study. The mitochondria-mediated pathway and death-receptor-mediated pathway will converge eventually to a common downstream pathway through caspase-3, which mediates the morphological and biochemical functions. In this study, caspase-3 was expressed differently on 316L SS and HNNF SS substrates, demonstrating their different effects on HUVECs growth. These data suggest that the apoptotic behavior of HUVEC on 316L SS substrate results from the exogenous pathway.

It should be kept in mind that cell proliferation, apoptosis and autophagy, and many other cellular processes are taking place simultaneously, and are linked with each other through a more complicated cross-talk network. Thus, one pathway might be a part of the whole inter-regulation process. These results in the present study could lead to a hypothesis that the high proliferation rate over apoptotic rate of 316L SS may contribute to the hypercellular restenosis after stent implantation. Previous study already showed that the imbalance between proliferation and apoptosis led to hypercellular neointima formation after stent implantation, and the apoptotic activity reached peak at day 7 and then decreased thereafter [36]. The dominance of proliferation over apoptosis may partly explain the great cellularity of early neointima.

### Autophagosic behavior of HUVECs

Autophagy is a biological event which provides a cell survival advantage under nutrient deprivation or other stress conditions. Atg5 has been considered as one of the components of the basic autophagic machinery. It participates in the ubiquitin-like conjugation systems that are essential for elongation of the pre-autophagosomal structures. Atg5 will be covalently linked to Atg12, an ubiquitin-like protein, and this linkage is facilitated by Atg7 and Atg10. The Atg12–Atg5 dimer then binds with Atg16L, forming a large multi-protein complex [37].

Since both autophagy and apoptosis are important cellular events involved in the cell development and cell proliferation, a crosstalk between these two pathways has been received a great attention [38]. Apoptosis and autophagy are not mutually exclusive. They have been shown to act in synergy and to count on each other [37]. Apoptotic regulators such as caspase-8 and FADD-like apoptosis regulator can regulate autophagy, and equally important, proteins such as Atg5 involved in autophagy can also induce caspase activation by interacting with the adaptor protein FADD, a component of the extrinsic apoptosis pathway [38], [39], [40], [41].

Quantitative RT-PCR analysis showed that three important autophagic regulators, ATG5, ATG7 and ATG12, were overexpressed in HUVECs grown on the 316L SS substrate, indicating that the autophagic pathway was fully activated (Fig. 7b). These results also mean that HUVECs must be under a stressed condition, probably due to the presence of nickel. In contrast, these three genes of HUVECs were expressed just at the basal level in the case of HNNF SS substrate. It might be the hint that although proliferations of the HUVECs on 316L SS and HNNF SS substrates are faster than that of the control, they might utilize different pathways (mechanisms) to stimulate the cells growth. This interesting phenomenon needs a further investigation.

### Effect on Cell Cycle of HUVECs

A cell needs to go through the G0/G1 phase, S phase and G2 phase, and is divided into two daughter cells finally in the mitosis phase. It has been known that cell cycle is modulated by many regulatory factors like cyclin proteins and cyclin-dependent kinases (CDKs). Thus, these cyclin proteins demonstrate a cycle-dependent expression profile. Cyclin Ds stimulate the cell entry into a new cell cycle at the end of the G1 phase and the activity of cyclin D1 is required throughout the G1 phase [42]. Cyclin Ds or Cyclin Es initiate DNA replication and centrosome duplication mainly, and their levels regulate the cells to enter into the S phase. Cyclin As are responsible for stimulating DNA replication, promoting some mitotic events, and preventing DNA re-replication, thus, the levels of cyclin As remain high throughout the S phase, the G2 phase and early mitosis [43].

In this study, qRT-PCR analysis showed that the expression levels of cyclin A, E and D of HUVECs on HNNF SS substrate were higher than those on 316L SS substrate (Fig. 7c), indicating that HUVECs on 316L SS might initiate the G1 phase earlier than that on HNNF SS. This interpretation is consistent with the results of Fig. 5 where HUVECs on 316L SS progressed slightly faster than those on HNNF SS. We assessed the influences of the cyclin proteins on the proliferations of HUVECs cultured on 316L SS and HNNF SS substrates, and correlated these changes with the cell proliferation behavior. To our knowledge, this is the first attempt to interpret the cellular response of cells to a biomaterial at the molecular level. In conjunction with the studies of cell apoptosis and autophagy, it might be a new approach to understand the regulatory mechanism underlying the cell adhesion, proliferation and migration on different biological compatible materials. Moreover, during cell progression, cyclins, CDKs and CKIs (cyclin-dependent inhibitors) were cooperated together to regulate the cell cycle. Therefore, better understandings for the cell regulation pathways and long-term effects of 316L SS and HNNF SS on the cellular response and thus the influence on ISR are extremely important and further investigations are being conducted.

### Effects of Nickel Ions

Although 316L SS has been widely used as a biomaterial for stent surgery, there are still concerns about its negative effect on human health, toxic, allergic or carcinogenic effects [5], [44]. Recent studies of microarray reported the molecular mechanism of the cell response to Ni^2+^ [5], [45], and the results suggested that the exposure of cells to Ni^2+^ may evoke a series of cellular responses to hypoxia by regulating hypoxia-inducible gene expression and cause irreversible DNA damage. The analysis of cell cycle pathway showed that DNA replication in S phase could be inhibited by Ni^2+^ [5]. In addition, Ni^2+^ can impair cell functions by inhibiting cell differentiation through inducing cell apoptosis, affecting cell development and influencing cholesterol metabolism [45].

In previous studies, 100 µM or even 200 µM Ni^2+^ concentration were used to assess the Ni^2+^ effects on cellular response [5], [45], which are much higher than the nickel content released from 316L SS. A smaller amount of nickel released in this setting could minimize its acute cell toxicity, which is in agreement with recent results from other studies by MTT test [30]. Meanwhile, we are aware of the fact that the 316L SS and HNNF SS are different not only in the Ni^2+^ content but also other elements, and the biological effects of other elements should not be simply ruled out. Therefore, the overall cellular response to the different substrates observed in this study needs further investigations.

### Conclusions

In this study, we investigated the cellular responses of HUVECs to two different types of stent materials: nickel-containing 316L SS and nickel-free HNNF SS. CCK-8 and flow cytometry analysis showed that 316L SS could activate the cellular apoptosis more efficiently and initiate an earlier entry into the S-phase of cell cycle than HNNF SS. To interpret the cellular behavior, we used qRT-PCR to analyze the gene expression profile of HUVECs. Quantitative RT-PCR results showed that the genes regulating cell apoptosis and autophagy were overexpressed on 316L SS. It might be attributed to the toxic effect of nickel ions. These data and conclusions lay a solid foundation to understand the ISR formation and to develop novel biomaterials for stent applications.
